# Retinal ganglion cell type-specific expression of synuclein family members revealed by scRNA-sequencing

**DOI:** 10.7150/ijms.95598

**Published:** 2024-05-27

**Authors:** Qingwen Yang, Lin Liu, Fang He, Wenna Zhao, Zhongqun Chen, Xiaotian Wu, Bilin Rao, Xin Lin, Fangyuan Mao, Jia Qu, Jun Zhang

**Affiliations:** 1 State Key Laboratory of Ophthalmology, Optometry and Visual Science, Eye Hospital, Wenzhou Medical University, Wenzhou, 325027, China.; 2 Laboratory of Retinal Physiology and Disease, School of Ophthalmology and Optometry, Wenzhou Medical University, Wenzhou, 325027, China.; 3 Alberta Institute, Wenzhou Medical University, Wenzhou, 325027, China.

**Keywords:** Retinal ganglion cells, synuclein family members, cell-type specificity, Single-cell RNA-sequencing, mouse retina

## Abstract

Synuclein family members (Snca, Sncb, and Scng) are expressed in the retina, but their precise locations and roles are poorly understood. We performed an extensive analysis of the single-cell transcriptome in healthy and injured retinas to investigate their expression patterns and roles. We observed the expression of all synuclein family members in retinal ganglion cells (RGCs), which remained consistent across species (human, mouse, and chicken). We unveiled differential expression of Snca across distinct clusters (highly expressed in most), while Sncb and Sncg displayed uniform expression across all clusters. Further, we observed a decreased expression in RGCs following traumatic axonal injury. However, the proportion of α-Syn-positive RGCs in all RGCs and α-Syn-positive intrinsically photosensitive retinal ganglion cells (ipRGCs) in all ipRGCs remained unaltered. Lastly, we identified changes in communication patterns preceding cell death, with particular significance in the pleiotrophin-nucleolin (Ptn-Ncl) and neural cell adhesion molecule signaling pathways, where communication differences were pronounced between cells with varying expression levels of Snca. Our study employs an innovative approach using scRNA-seq to characterize synuclein expression in health retinal cells, specifically focusing on RGC subtypes, advances our knowledge of retinal physiology and pathology.

## Introduction

Synucleins are a family of abundant presynaptic proteins, consisting of alpha- (α-Syn), beta- (β-Syn), and gamma-synuclein (γ-Syn), which are encoded by Snca, Sncb, and Sncg, respectively 1. α-Syn and β-Syn are primarily found in the central nervous system (CNS), including the retina 2-4, where they mainly localize in presynaptic terminals 4-6. On the other hand, γ-Syn is primarily found in the peripheral nervous system and retina ganglion cells (RGCs), and its expression in breast cancer serves as a marker for cancer progression 7, 8. In the CNS, the function of synuclein has been extensively studied. It plays a critical role in vesicle recycling at the presynaptic terminal, including SNARE complex formation 9, fusion pore dilation 10, and regulation of synaptic vesicle endocytosis 11 and so on. However, despite these investigations, the normal cellular function and expression pattern of synuclein in the retina remain poorly understood.

Synucleins exhibit high expression levels in the retina and optic nerve 2, 12, 13. Their expression patterns have been characterized using immunofluorescence (IF), revealing significant diversity among different cell types in the retina 2, 3, 14-16. For instance, α-Syn is mainly found in the inner plexiform layer (IPL), inner nuclear layer (INL), and the ganglion cell layer (GCL). 2, 17, 18. β-Syn is primarily localized in the GCL, IPL, INL, and the outer plexiform layer (OPL) 2, 19. On the other hand, γ-Syn is predominantly located in the GCL and nerve fiber layer 3, 12, 20. These findings confirm that all synuclein family members are concentrated in the GCL, which is where the majority of RGCs exist. This suggests that synucleins may play a role in transmitting visual signals from RGCs to higher visual centers. Although the expression of synuclein family members in the retina has been profiled using IF approach, these studies have provided limited insights into the molecular differences of synuclein expression among different RGC subtypes.

The classification of RGCs is diverse based on their morphology, molecular expression, and function 21, 22. Morphological types of RGCs are categorized according to soma size and dendritic stratification in the IPL, resulting in three main types: monostratified, bistratified, and diffusestratified 23, 24. In humans and primate animals, for example, 18 morphological types of RGCs have been reported 25. Functionally, RGCs are also classified into three subtypes based on their responses to light stimuli: ON RGCs, OFF RGCs, and ON-OFF RGCs 26, 27. Additionally, molecular studies have identified 46 clusters, leading to the subdivision of RGCs into various subclasses, such as α-RGCs, T-RGCs, F-RGCs, ipRGCs, ooDSGC, N-RGCs, and T5-RGCs, based on their transcriptome 28. Each of these defined RGC subtypes possesses specific attributes contributing to visual perception 26. Although previous evidence shows expression of all synuclein family members in RGCs 2, 3, 12, 17-20, 29, including some intrinsically photosensitive retinal ganglion cells (ipRGCs) and Brn3a/b positive RGCs, which subtypes of RGCs express different synuclein family members remains uncertain. Additionally, the expression patterns of β-Syn and γ-Syn in comparison to α-Syn remains unclear.

Various RGC types exhibit different susceptibility to injury 28, 30-32. Tran *et al.* 28 reported that all ipRGCs, ON-sustained (ON-S), and OFF-sustained (OFF-S) α-RGCs are resistant to cell death. Conversely, ooDSGCs are highly susceptible to injury, and OFF-transient α-RGCs show a vulnerability similar to ooDSGCs, while all N-RGC types are susceptible. Optic nerve crush (ONC) is an experimental procedure used to study the response of RGCs and their axons to injury, leading to the death of approximately 80% of RGCs within two weeks. Recent research has revealed that certain RGC types differ in their ability to survive following ONC. For instance, α-RGC types have a higher ability to survive axotomy than other RGC types in both mice 30 and cats 31 retinas. Synuclein family members are expressed in RGCs, but it remains unclear whether their expression significantly changes during ONC and whether they are susceptible to injury following ONC.

Technological advances, particularly high-throughput transcriptomic techniques, have granted us powerful tools at the single-cell level to unravel the patterns of neuron involvement and molecular changes in healthy and diseases 33, 34. Single-cell RNA sequencing (scRNA-seq) has been instrumental in identifying molecular differences among various retinal cell types, including RGCs, bipolar cells (BCs), and amacrine cells (ACs) 35-37.

In this study, we present a comprehensive profiling of synuclein family member expression at the single-cell level in human, mouse, and chicken retinal cell types. Additionally, we validate these findings in enriched RGCs, ACs, and BCs. Furthermore, we analyze the transcriptomic differences of synuclein family members among a purified RGC atlas. Finally, we investigate the molecular changes of synuclein family members in a diseased retina using an ONC model. Our work provides an extensive molecular atlas of synuclein family members in healthy and injured retinas, offering valuable insights into their function and dysfunction. This approach holds great potential for advancing our understanding of the role of synuclein family members and neurodegenerative diseases in retinal health and pathology.

## Materials and Methods

### Data acquisition and processing

Single-cell transcriptomic datasets for the retina of humans were obtained from Gene Expression Omnibus (GEO, https://www.ncbi.nlm.nih.gov/geo/) with the accession numbers: GSE147979. The dataset of Gautam *et al.* 38 were obtained from post-mortem human adult eyes of 6 donors ageing between 28 and 84 years old within 24 h after death. We extracted retinal cells for analysis, involving 15,978 cells from 4 donors. Single-cell transcriptomic datasets for the retina of mice were obtained from GEO with the accession numbers: GSE63472. The dataset of Macosko *et al.* 33 were obtained from C57BL/6 wild-type P14 mouse retinas, involving 6237 cells from 7 mice. Single-cell transcriptomic datasets for the retina of chicken were obtained from GEO with the accession numbers: GSE159107. The dataset of Yamagata *et al.* 39 includes 35,026 retinal cells from embryonic day 18 chick retina. For most of the analysis, the GSE137400 dataset were obtained from GEO, containing RGC cells from adult mice ageing 6 to 20 weeks, was used. This dataset includes an RGC atlas with 35,699 cells from three biological replicates and an RGC dataset following optic nerve crush with 76,646 cells from 32 mice 28. It is important to note that all cell clusters in this study refer to previous studies conducted 28, 33, 38, 39. All the downstream analysis of scRNA-seq data were conducted using the R package, Seurat (version 4), following the code that previous studies provided 28, 33, 38, 39.

### Differential expression analysis

The differentially expressed genes (DEGs) were conducted using the function of R package 'Seurat' and the 'FindAllMarkers'. We performed a separate analysis to assess the heterogeneity of expression between samples from the RGCs expressing Snca (RGCs-Snca^+^) group and not expressing Snca (RGCs-Snca^-^) group. Adjusted p value (p_val_adj, < 0.05) was used to determine the difference was statistically significant.

### Functional Annotation of Genes

The R package 'clusterProfiler' were employed for Gene Ontology (GO) enrichment analysis. The p-values were adjusted by the 'BH' method. The reference database utilized for the analysis was 'org.Mm.eg.db', which is specific to mice. Top 100 DEGs were selected for GO analysis. Statistical significance was determined by an adjusted p-value of less than 0.05 for enrichment results. The gene coincidence rate was defined as the overlap ratio between the enriched genes to the background genes in each functional term. AutoAnnotate in Cytoscape (v3.8.0) (https://cytoscape.org/) 40 was employed to visualize and cluster the enrichment results.

### Cell type enrichment

We quantify the enrichment of cell types 41 across different conditions, such as RGCs-Snca^+^ and RGCs-Snca^-^ to remove the impact of sample variations. If the value is greater than 1, it indicates that a particular cell type is enriched in a specific condition (e.g., RGCs-Snca^+^ or RGCs-Snca^-^).

### Cell-cell communication analysis

The R package CellChat (version 1.0.0) was used to infer and quantify the cell-cell communication among cell types 42. This R package involved integrating single-cell expression profiles, signaling from ligands, receptors and cofactors to infer the probability of cell-cell communication. The functions 'computeCommunProb' and 'filterCommunication' were used to identify the potential interactions at the ligand-receptor level. The function 'computeCommunProbPathway' were used to analyze the cellular communication network at the signaling pathway level.

### Animals and tissue preparation

C57BL/6J mice were purchased from GemPharmatech (Nanjing, China). All animal experiments and procedures were permitted by the Animal Care and Ethics Committee at Wenzhou Medical University in accordance with the ARVO guidelines. All mice were housed on a 12-hour (h) light/12-h dark cycle and given a standard chow diet. Tissue preparation was done as described in previous study 17, 43. Briefly, all mice were anesthetized, decapitated and enucleated, the anterior segment and vitreous were removed from the eyes. We then fixed the posterior eyecups in 4% paraformaldehyde in 0.1 M phosphate buffer (PB, pH 7.4) for 25-30 minutes at room temperature. The eyecups were then washed 4-5 times after fixation in 0.1 M phosphate-buffered saline (PBS, pH 7.4) for ten minutes each. Subsequently, eyecups were dehydrated with graded sucrose solution. After embedding, the eyecups were sectioned in a cryostat (transverse sections of 25 μm thickness), and mounted onto glass slides.

### IF staining

IF staining was performed as described previously 17, 43. Briefly, the cryosections were washed with PBS and blocked in PBS containing 5% normal donkey serum (NDS; Sigma, St. Louis, MO) for 2 hours at room temperature (RT), incubated in primary antibodies in 2% NDS plus 1% bovine serum albumin (BSA, Sigma) with 0.3% Triton X-100 at 4 ºC overnight and then incubated with secondary antibody at RT for 2h. The primary antibodies used in this study were summarized in Table 1, including the sources, cell types, reference, and working dilutions. Whole mount retinas were incubated with primary antibody for 4 days and with secondary antibody for 1 days in the dark at 4 ºC. Subsequently, whole mount retinas and sections were washed in PBS and coverslipped with DAPI.

### ONC Model

The adult mice were deeply anesthetized and placed under an operating microscope. Following the method described previously 28, 44, the left eye's optic nerve was exposed within the orbit after the blunt separation of the conjunctival sac using two surgical forceps around the lateral canthus. Carefully, the exposed optic nerve was crushed about 0.5-2 mm behind the optic disc using pointed self-closing forceps (5X.SA, ideal-tek, Switzerland) for 20 seconds, ensuring that the retinal blood vessels and blood supply were not damaged. After the surgery, eye ointment was used to protect the cornea. All procedures were carried out under sterile conditions, and no postoperative infections were observed.

### Confocal image analysis and quantification

Retina sections were visualized with a Zeiss LSM900 confocal microscope through 20×/0.8 objective and 40×/1.2 water immersion objective. The Z-stacked images with a thickness of 15-25 μm and a vertical resolution of 1.5 μm were taken for soma analysis. Adobe Photoshop 6 was used to adjust the brightness and the contrast of the final images.

For quantitative analysis of α-Syn immunoreactive (IR) RGCs in the GCL, double labeling of RBPMS with α-Syn was performed in vertical sections using 20×/0.8 objective. A total of 407 α-Syn-IR RGCs were analyzed on 22 sections from four animals in the control group, and 164 α-Syn-IR RGCs were analyzed on 21 sections from five animals in the ONC group, which covered the whole retinal sub-regions (nasal, temporal, dorsal and ventral).

For quantitative analysis of α-Syn-IR ipRGCs in the GCL, double labeling of melanopsin (a biomarker for ipRGCs) with α-Syn was performed on vertical sections using the 40×/1.2 water immersion objective. A total of 88 ipRGCs IR RGCs were analyzed on 7 sections of four animals in the control group, and 62 ipRGCs IR RGCs were analyzed on 6 sections of four animals in the ONC group, covering the whole retinal sub-regions (nasal, temporal, dorsal and ventral).

### Statistical analysis

The quantification data were presented as means ± SD. Statistical analysis for all bioinformatic analysis were performed in R software (Version 4.1.2). P values < 0.05 was considered significant. Statistical analysis for all experiment were accomplished with GraphPad software (Prism 7.0; San Diego, CA, USA). Comparison of the results between control group and ONC group was performed with two-tailed Student's t-test. Comparisons of the results among different groups were accomplished with one-way ANOVA with Tukey's multiple comparison tests.

## Results

### Cross-species expressions of synuclein family members revealed by scRNA-seq

**Figure 1:**
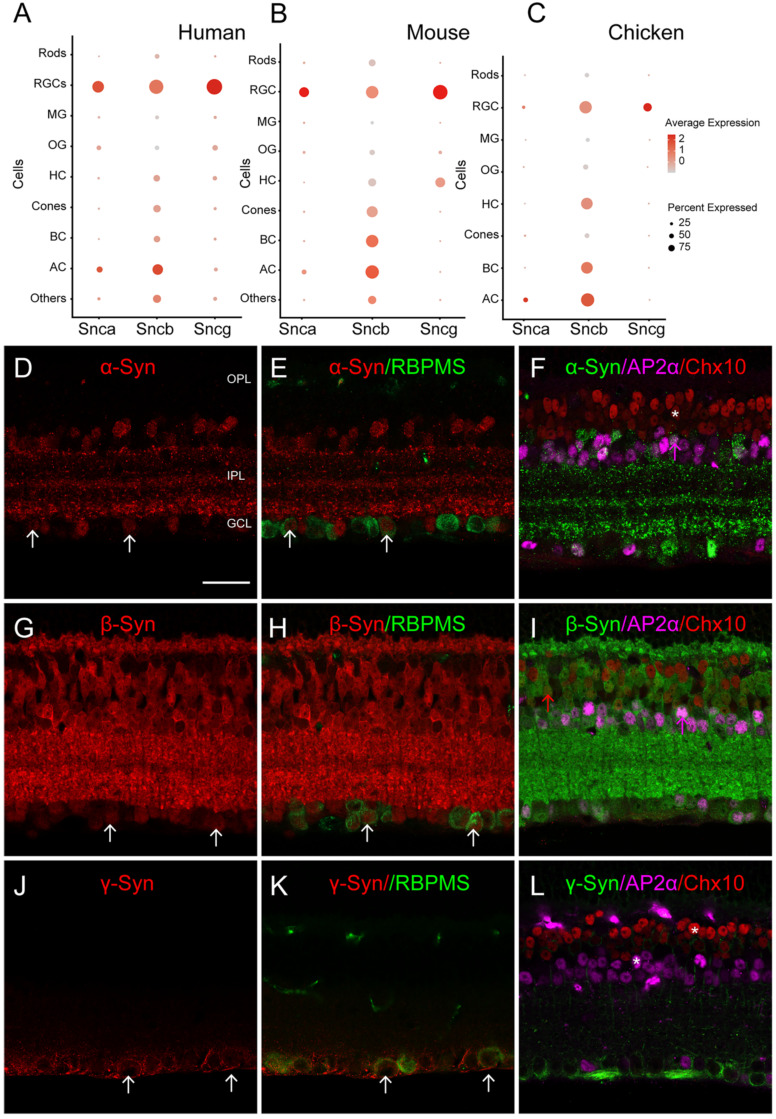
** The expression pattern of synuclein family members in the retina of various species. (A-C)** Single-cell RNA sequencing (scRNA-seq) reveals the distribution of synucleins expression across retinal cell types, including rod photoreceptors (Rods), retinal ganglion cells (RGCs), Müller glia (MG), oligodendrocytes (OG), horizontal cells (HCs), cone photoreceptors (Cones), bipolar cells (BCs), amacrine cells (ACs).** (A)** Dotplot of synuclein family members expression (Snca, Sncb and Sncg) in various retinal cell types of normal human retina. **(B)** Dotplot of synuclein family members expression (Snca, Sncb and Sncg) in various retinal cell types of normal mouse retina. **(C)** Dotplot of synuclein family members expression (Snca, Sncb and Sncg) in various retinal cell types of normal chicken retina. For each cell type, the size of each dot (pct.exp) indicates the percentage of cells in which the gene was detected, and its color indicates the average transcript count in the cells that expressed it (avg.exp.scale). **(D-L)** Immunohistochemical analyses of synuclein expression in BCs, ACs and RGCs in the mouse retina. **(D-E)** Double labeling of α-Syn **(D)** and RBPMS **(E)**, a biomarker for RGCs. A micrograph showed the colocalization of α-Syn and RBPMS (white arrows). **(F)** Triple labeling of α-Syn with AP2α and Chx10, biomarkers for ACs and BCs, respectively. α-Syn was colocalized with AP2α (pink arrows), while not colocalized with Chx10 (white stars). **(G-H)** Double labeling of β-Syn **(G)** and RBPMS **(H)**. A micrograph showed the colocalization of β-Syn and RBPMS (white arrows). **(I)** Triple labeling of β-Syn with AP2α and Chx10. β-Syn was colocalized with AP2α and Chx10 (pink arrows and red arrows). **(J-K)** Double labeling of γ-Syn **(J)** and RBPMS **(K).** A micrograph showed the colocalization of γ-Syn and RBPMS (white arrows). **(L)** Triple labeling of γ-Syn with AP2α and Chx10. γ-Syn was not colocalized with AP2α and Chx10 (white stars). Scale bars: 40 μm **(D-L)**.

We utilized bioinformatics analysis of scRNA-seq data to investigate the expression of synuclein family members (Snca, Sncb, and Sncg) in the retina. By classifying retinal cell types and analyzing their gene expression profiles, we found cell-type specific expression patterns of Snca, Sncb, and Sncg. In the human retina (Fig. 1A), Snca was predominantly expressed in RGCs and ACs, while Sncb showed high expression in all retinal neurons. Sncg, on the other hand, was specifically detected in RGCs. To assess conservation of expression patterns, we performed a comparative transcriptome analysis across species. In mice (Fig. 1B), Snca expression was highest in RGCs and ACs, while absent in BCs. Sncb was expressed in all retinal neurons including RGCs, ACs, and BCs, with relatively even distribution in non-neuronal retinal cells. Sncg showed high expression in RGCs and limited expression in ACs and BCs. Similarly, the expression pattern of Snca, Sncb, and Sncg in chicken retina resembled that of the human retina (Fig. 1C). These findings indicate a conserved expression pattern of synuclein family members in the retina across different species.

To confirm the expression of synuclein family members in retinal cell types, we performed IF on mouse retinal sections. We observed that α-Syn, encoded by Snca, colocalized with RBPMS (RGC marker) and AP2α (AC marker), confirming the expression of α-Syn in RGCs and ACs (Fig. 1D-F). However, α-Syn did not colocalize with Chx10, a BC marker, indicating its absence in BCs. These IF findings were consistent with the scRNA-seq data. Additionally, β-Syn, encoded by Sncb, was detected in RGCs, ACs, and BCs based on the scRNA-seq data. IF revealed colocalization of β-Syn with RBPMS, AP2α, and Chx10 (Fig. 1G-I), supporting its expression in these cell types. Furthermore, γ-Syn, encoded by Sncg, was specifically detected in RGCs according to the scRNA-seq data. IF showed colocalization of γ-Syn with RBPMS (Fig. 1J-K), while it did not colocalize with AP2α and Chx10 (Fig. 1L), confirming its specific expression in RGCs. These IF results provide further validation of the expression patterns observed in the scRNA-seq data.

### Unique gene expression profiles of Snca in RGCs populations compared to Sncb and Sncg

RGCs exhibit diverse morphology, molecular expression, and function, and previous studies have identified 46 molecularly distinct clusters within the RGC population 28. Above Single-cell analysis found that all members of the synuclein family were highly expressed in RGCs across species (Figure 1A-C), suggesting that synucleins may play a role in transmitting visual signals from RGCs to higher visual centers. However, due to these datasets were derived from cell suspension throughout the retina, where the number of rods and cones detected accounted for the majority, while RGC accounted for only a small fraction of the cell population (1-2%).

**Figure 2:**
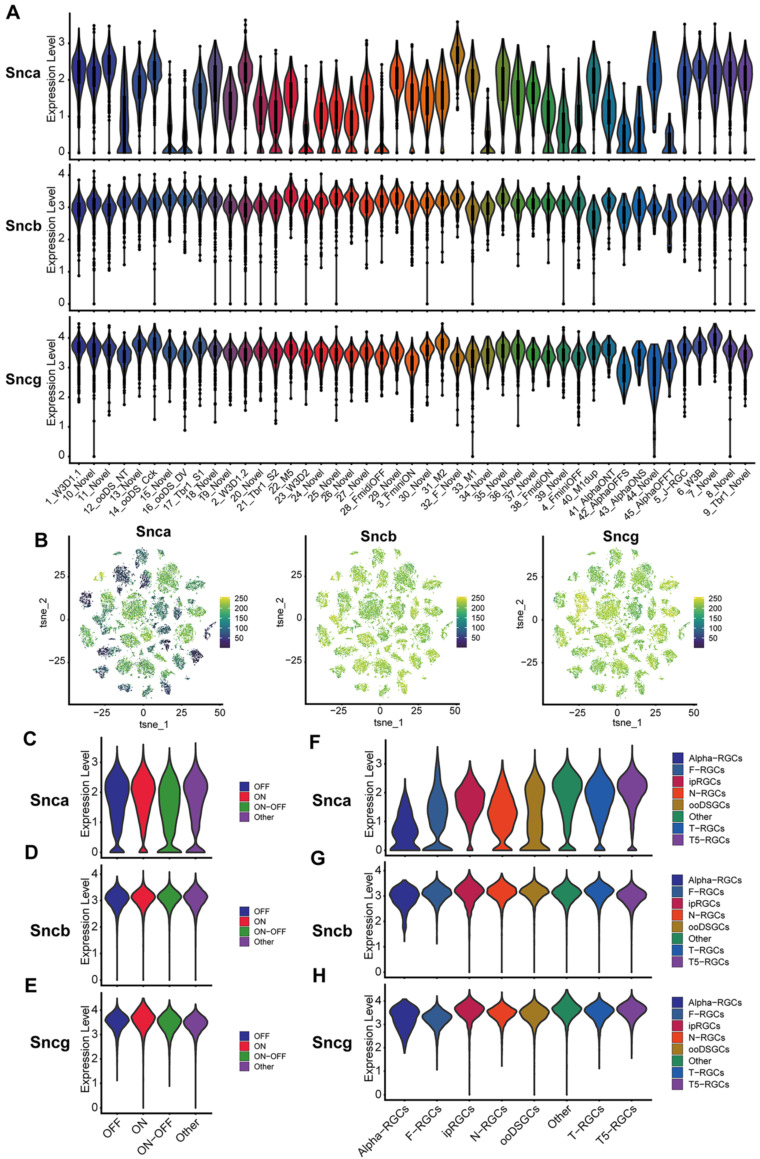
**scRNA-seq analysis of synuclein family members expression in various RGC subtypes. (A)** Violin plots depicting the expression levels of synuclein family members (Snca, Sncb, and Sncg) in 46 RGCs clusters. The y-axis represents the gene expression, while the x-axis indicates the different RGC clusters. **(B)** t-SNE plots illustrating the expression patterns of Snca, Sncb, and Sncg across all RGCs clusters. Each dot represents an individual RGC cell. **(C-E)** Violin plots displaying the expression levels of synuclein family members in three functional types of RGCs: ON, OFF, and ON-OFF RGCs. **(F-H)** Violin plots showing the expression levels of synuclein family members in eight classified subtypes of RGCs.

Additionally, due to the limitations of sequencing technology and the total number of RGCs collected, we investigate the expression of Snca, Sncb, and Sncg in specific subtypes of RGCs by analyzing purified mouse RGC scRNA-seq data (details in Methods). We examined the distribution of Snca, Sncb, and Sncg among 46 clusters to determine if any specific subtypes showed enriched expression of the synuclein family members. The analysis revealed that Snca exhibited differential expression across distinct clusters, while Sncb and Sncg displayed homogeneous expression across all clusters (Fig. 2A). Snca showed low expression in a small number of clusters but was highly expressed in most clusters. In contrast, Sncb and Sncg exhibited high expression levels in almost all clusters. The characteristic expression patterns of Snca, Sncb, and Sncg were visualized using tSNE plots, which showed that cells with low Snca expression tended to cluster together within specific RGC subtypes (Fig. 2B). Conversely, cells with high expression levels of Sncb and Sncg were distributed throughout the different clusters. To further examine the expression of synuclein family members in functional RGC subtypes, we utilized a classification of RGCs into three major types: ON, OFF, and ON-OFF RGCs, which respond to specific light stimuli 26. The analysis showed that Snca, Sncb, and Sncg exhibited homogeneous expression across all three RGC functional subtypes (Fig. 2C-E). The expression levels of Snca were relatively low and broadly distributed (Fig. 2C), whereas Sncb and Sncg displayed relatively high and concentrated expression levels (Fig. 2D, E).

To understand the expression profiles of Snca, Sncb, and Sncg in RGC subtypes, we referred to a classification system proposed by Tran *et al.* 26. This classification categorized RGCs into eight subtypes based on their transcriptomes, including α-RGCs, T-RGCs, F-RGCs, ipRGCs, ooDSGCs, T5-RGCs, N-RGCs, and others. Building upon this classification, we analyzed the expression of Snca, Sncb, and Sncg across these RGC subtypes, revealing cell-type specific expression patterns. Among the RGC subtypes, α-RGCs exhibited the lowest levels of Snca expression, whereas other RGC subtypes displayed relatively higher expression levels (Fig. 2F). In contrast, Sncb and Sncg showed greater expression across all RGC subtypes (Fig. 2G, H). These findings suggest that Sncb and Sncg exhibit similar expression patterns, while Snca demonstrates distinct gene expression profiles in different populations of RGCs. These scRNA-seq data provide valuable insights into the differential expression of synuclein family members in specific RGC subtypes, contributing to our understanding of their functional roles in different populations of RGCs.

**Figure 3:**
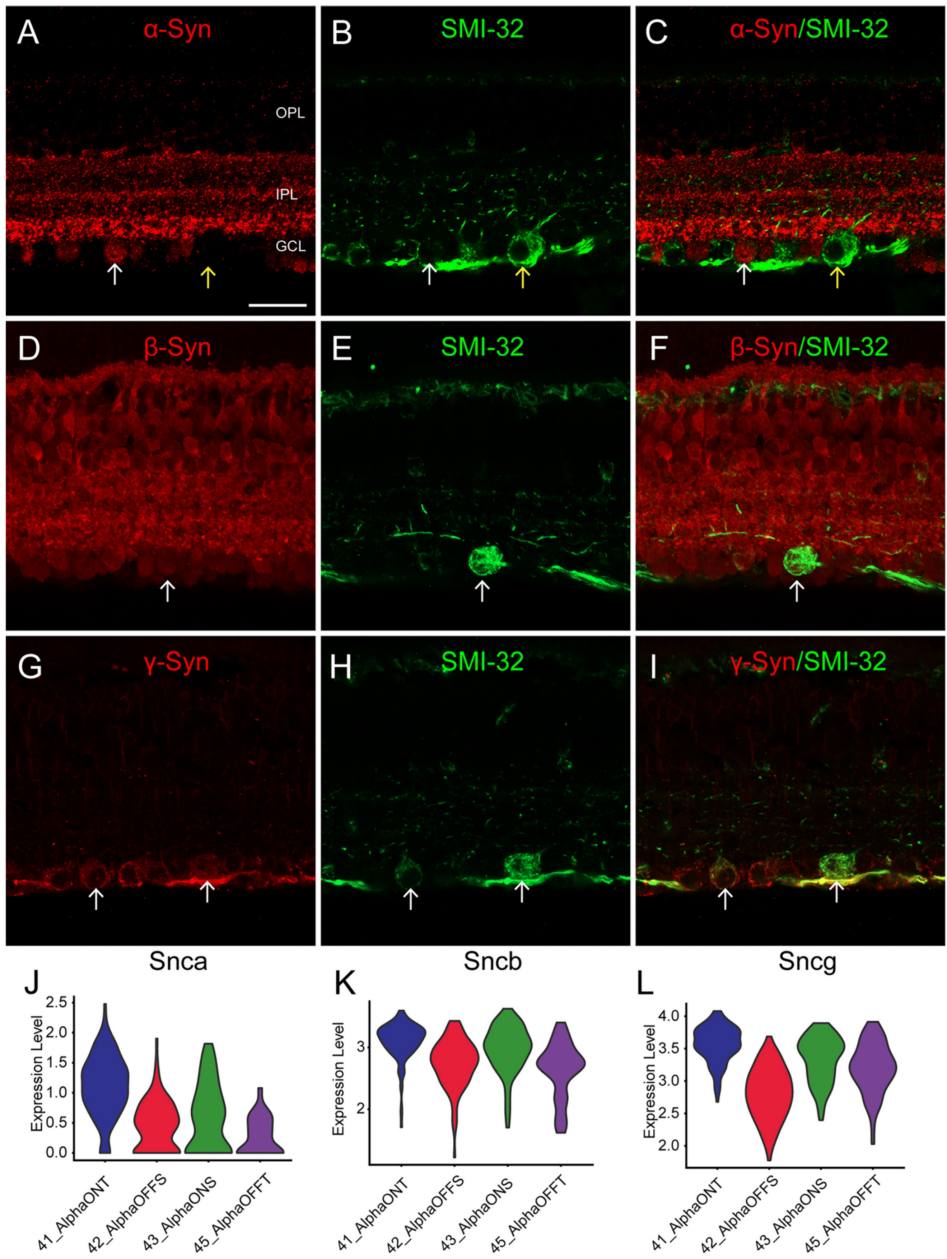
** Identification of synucleins expression in α-RGCs. (A-I)** Immunohistochemical analyses of synucleins expression in α-RGCs in vertical sections of the mouse retina. **(A-C)** Double labeling of α-Syn (A) and SMI-32 **(B)**, a biomarker for α-RGCs. The micrograph shows the colocalization of α-Syn and SMI-32 (white arrows). **(D-F)** Double labeling of β-Syn **(D)** and SMI-32 **(F)**. The micrograph shows the colocalization of β-Syn and SMI-32 (white arrows).** (G-I)** Double labeling of γ-Syn **(G)** and SMI-32** (I)**. The micrograph shows the colocalization of γ-Syn and SMI-32 (white arrows). **(J-L)** Distribution of synucleins expression in α-RGCs subtypes delineated by single-cell transcriptomics. Violin plots depicting the expression levels of Snca **(J)**, Sncb **(K)** and Sncg **(L)** in four α-RGCs subtypes (On-sustained α-RGCs, On-transient α-RGCs, Off-sustained α-RGCs, and Off-transient α-RGCs). Scale bars: 40 μm **(A-I)**.

To validate the expression patterns of synuclein family members in α-RGCs identified through scRNA-seq, we performed IF using the SMI-32 antibody, which is commonly used to label α-RGC populations with large, strongly labeled somas. IF results demonstrated that strongly SMI-32 IR somas were not colocalized with α-Syn IR somas in the GCL (Fig. 3C, yellow arrow). However, weakly SMI-32 IR somas were colocalized with α-Syn IR somas (Fig. 3C, white arrow), consistent with the scRNA-seq data indicating low expression of Snca in α-RGCs. Conversely, the scRNA-seq data showed high expression of Sncb and Sncg in α-RGCs. Accordingly, all SMI-32-positive RGCs displayed colocalization with β-Syn and γ-Syn IR somas (Fig. 3D-I), respectively. These findings suggest that α-RGCs express high levels of β-Syn and γ-Syn, while exhibiting low expression of α-Syn.

It should be noted that α-RGCs can be further classified into four subtypes based on their light response properties (On-sustain, On-transient, Off-sustain, and Off-transient). However, it is challenging to identify functional α-RGC subtypes using IF alone.

To address this, we extracted α-RGCs from the scRNA-seq data and categorized them as On-sustained α-RGCs (ON-S), On-transient α-RGCs (ON-T), Off-sustained α-RGCs (OFF-S), and Off-transient α-RGCs (OFF-T). We observed that Snca was highly expressed in ON-T α-RGCs and displayed lower expression in the other three α-RGC subtypes (Figure 3J). Additionally, Sncb and Sncg exhibited relatively low expression in OFF α-RGCs (OFF-S and OFF-T) and relatively high expression in ON α-RGCs (ON-S and ON-T) (Fig. 3K, L). These findings provide further support for the differential expression of synuclein family members in distinct α-RGC subtypes, contributing to our understanding of their functional roles in the visual processing pathway.

### Heterogeneity of RGC subtypes based on Snca expression levels

**Figure 4:**
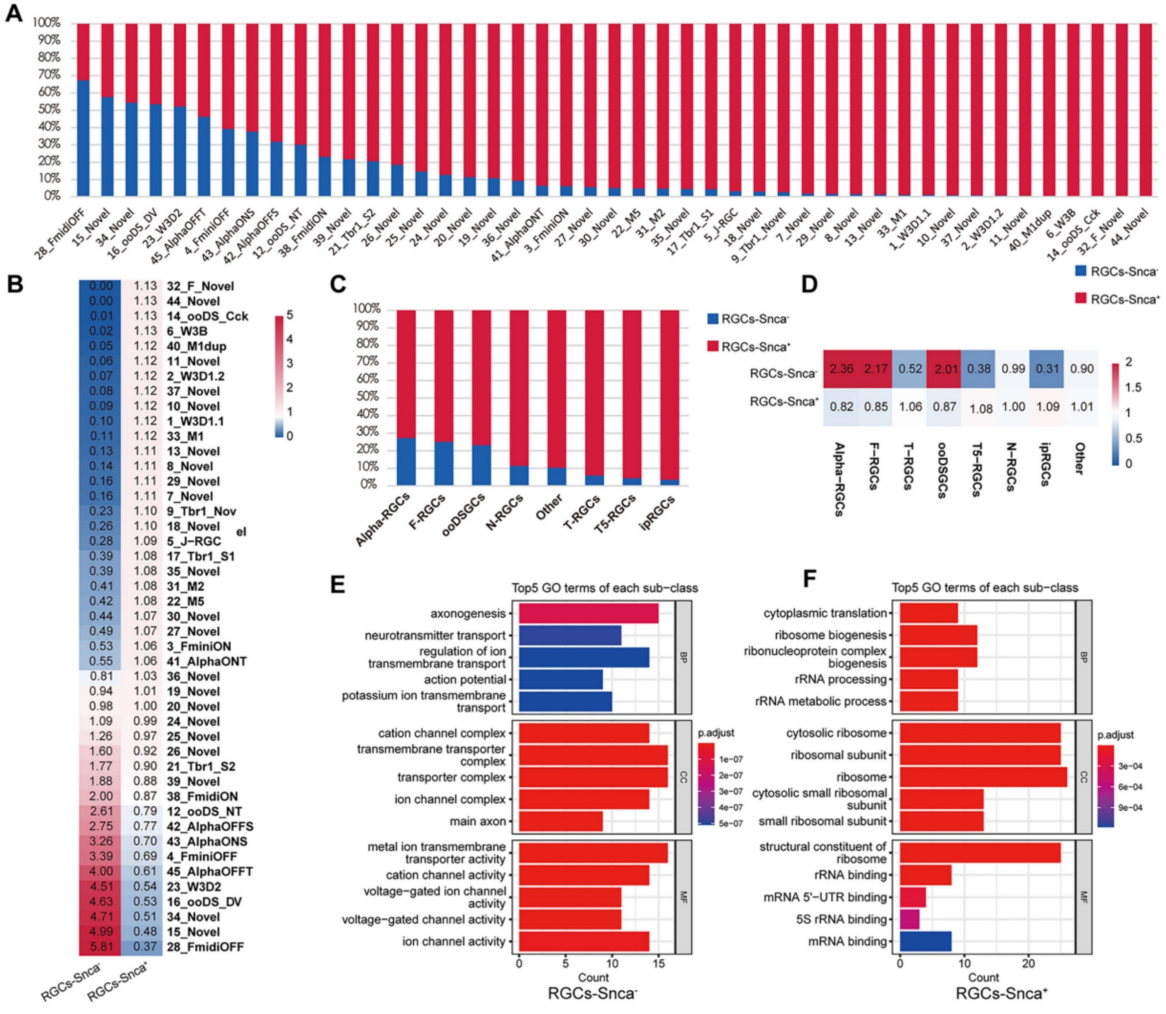
** scRNA-seq reveals the differential expression of Snca in RGCs subtypes. (A, C)** Proportion of cells expressing Snca (RGCs-Snca+) and not expressing Snca (RGCs-Snca-) in 46 RGCs clusters and 8 classified RGCs subtypes. **(B, D)** Heatmap displaying the relative enrichment of each RGCs subtype in RGCs-Snca+ and RGCs-Snca-. The values larger than 1 indicate that the corresponding cell type is enriched in RGCs-Snca+, otherwise in RGCs-Snca-. **(E, F)** Gene Ontology (GO) enrichment analysis of the top 100 differentially expressed genes (DEGs) that are specific to RGCs-Snca+ **(E)** and RGCs-Snca- **(F)**.

As mentioned earlier, the expression of Snca showed differential patterns across the 46 molecularly distinct clusters (Fig. 2A). Utilizing scRNA-seq data, we quantified the fraction of RGCs expressing Snca (RGCs-Snca^+^) and not expressing Snca (RGCs-Snca^-^) within each RGC subtype. Substantial variations were observed in the cell fractions for each subtype. We further ranked the RGC subtypes based on the proportion of RGCs-Snca^-^ (Fig. 4A), which ranged from approximately 0% to 67.31%. Analyzing the cell proportion among each RGC subtype, we found that the majority of RGC subtypes were dominated by RGCs-Snca^+^. However, in subtypes such as C28-FmidiOFF (a subclass of the F-RGCs defined by the expression of the transcription factors *Foxp2+*), C15-Novel (an undefined new RGC subtype), C34-Novel (an undefined new RGC subtype), C16-ooDS-DV (a subclass of the ooDSGCs specifically expressing the dorsal and ventral-preferring type marker, *Co125a1* ), and C23-W3D2 (a subclass of the T5-RGCs defined by the integral membrane protein *Tusc5+*), the proportion of RGCs-Snca^-^ was higher than those RGCs-Snca^+^, indicating that these subtypes predominantly consist of cells without Snca expression (Fig. 4B). After accounting for potential influencing factors, such as tissue sampling bias or unclear cell numbers, it was evident that these five RGC subtypes were indeed enriched in RGCs-Snca^-^. Interestingly, all subtypes of ipRGCs (C40-M1dup, C33-M1, C31-M2, and C22-M5) exhibited enrichment in RGCs-Snca^+^ (Fig. 4C, also see Fig. 10H in 17).

Furthermore, ON-T α-RGCs (C41) showed enrichment in RGCs-Snca^+^, whereas ON-S(C43), OFF-S(C42), and OFF-T (C45) α-RGCs exhibited enrichment in RGCs-Snca^-^, indicating distinct Snca expression patterns among α-RGC subtypes. Additionally, we observed that α-RGCs, T-RGCs, and ooDSGCs were significantly enriched in RGCs-Snca^-^, while F-RGCs, ipRGCs, T5-RGCs, and N-RGCs were significantly enriched in RGCs-Snca^+^ (Fig. 4C-D). These findings highlight the cellular heterogeneity and diversity in RGCs associated with different levels of Snca expression. To explore further into potential functional differences related to Snca expression levels, we conducted GO enrichment analysis of DEGs between RGCs-Snca^+^ and RGCs-Snca^-^ (Fig. 4E-F). The GO enrichment analysis uncovered that the function of RGCs-Snca^+^ were associated with axonogenesis, while the function of RGCs-Snca^-^ were related to cytoplasmic translation and ribosomal binding processes. These results jointly suggest that RGCs with varying Snca expression levels play distinct roles in visual processes.

### Molecular changes of synuclein family members after ONC

**Figure 5:**
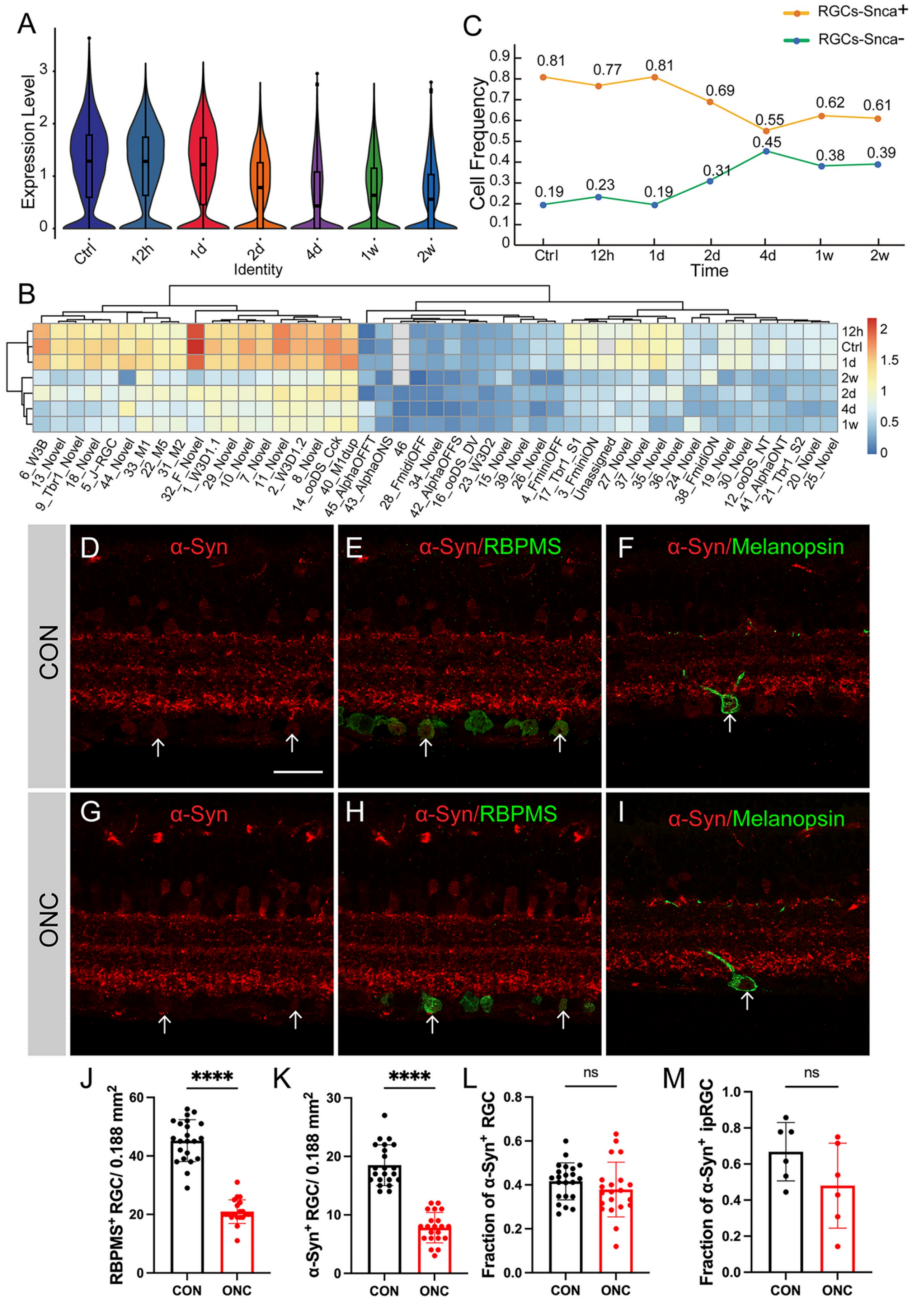
** Molecular changes of Snca after ONC. (A-C)** The decrease of Snca on mRNA level after ONC is delineated by scRNA-seq. **(A)** Violin plots reveal the decrease in the expression level of Snca after injury. **(B)** Heatmap displays the change in Snca expression among 46 RGCs clusters at each time point after ONC. **(C)** The change in the proportion of RGCs-Snca+ and RGCs-Snca- at each time point after ONC is depicted. **(D-M)** Immunohistochemical analyses of the susceptibility of α-Syn-positive RGCs and α-Syn-positive ipRGCs after ONC in vertical sections of the mouse retina. **(D- I)** Retinas were stained with RBPMS (a marker for total RGCs), α-Syn (α-Syn-positive cells), and markers specific to ipRGCs from control and ONC retinas. **(J)** Quantification of the number of RBPMS+ cells in control and ONC retinas. **(K)** Quantification of the number of colocalization of α-Syn+ and RBPMS+ cells in control and ONC retinas. **(L)** The fraction of α-Syn-positive RGCs in control and ONC retinas. **(M)** The fraction of α-Syn-positive ipRGCs in control and ONC retinas. Data were obtained by analyzing 21-22 central retinal slices derived from 4 to 5 animals in each group. ***P<0.001. Scale bars: 40 μm **(D-I).**

**Figure 6:**
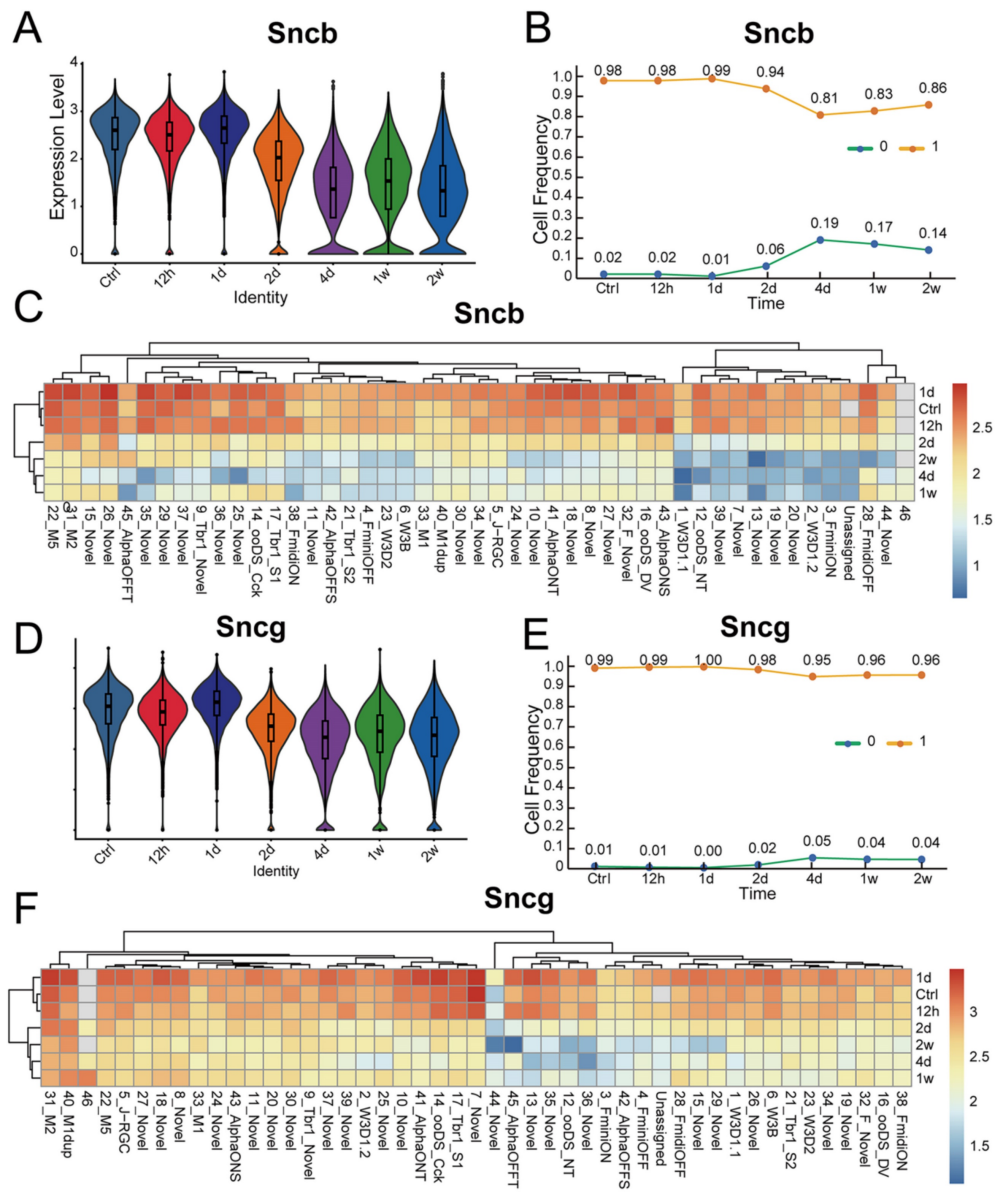
** Molecular changes of Sncb and Sncg after ONC. (A, D)** Violin plots reveal the decrease of the expression level of Sncb and Sncg after ONC.** (B)** The change in the proportion of cells expressing Sncb (RGCs-Sncb+) and not expressing Sncb (RGCs-Sncb-) at each time point after ONC. **(C, F)** Heatmaps display the change in expression of Sncb and Sncg among 46 RGC clusters at each time point after ONC. **(E)** The change in the proportion of cells expressing Sncg (RGCs-Sncg+) and not expressing Sncg (RGCs-Sncg-) at each time point after ONC.

Numerous studies have demonstrated that various types of RGCs exhibit different levels of resilience to injury 28. To investigate the susceptibility of synuclein family members to injury, we utilized an ONC model to induce RGC injury. Here, we analyzed the expression differences of Snca, Sncb, and Sncg in RGCs following ONC at multiple time points (12h, 1d, 2d, 4d, 1w, 2w), using a publicly available RGC scRNA-seq database. The expression of Snca showed no significant changes at 12h and 1d after ONC, followed by a significant decrease at 2d after ONC, and maintained at 4d after ONC (Fig. 5A). A notable shift in Snca expression was observed between control, 12h, and 1d after ONC, compared to 2d, 4d, 1w, and 2w after ONC (Fig. 5B). Specifically, Snca expression remained unchanged at 1d after ONC but dramatically decreased at 2d after ONC. Additionally, the cell fraction of RGCs-Snca^+^ and RGCs-Snca^-^ also changed at each time point after injury (Fig.5C). Furthermore, the expression levels of Sncb and Sncg were also decreased following ONC (Fig.6A, C, D, F). However, the proportion of cells expressing Sncg (RGCs-Sncb^+^) was almost unaltered after injury, while those expressing Sncg (RGCs-Sncb^+^) were slightly decreased (Fig. 6B, E). These findings suggest dynamic alterations in the expression of Snca, Sncb, and Sncg in response to ONC-induced injury. We also validated the transcriptomic data through IF (Fig. 5D-E, G-H) and revealed that the number of colocalization of α-Syn with RBPMS was decreased, further elucidating the response of synuclein family members to injury *in vivo*. Our findings shed light on the molecular changes in RGCs after injury and provide insights into the role of synuclein family members in the process.

To assess whether α-Syn expression is susceptible to injury, we analyzed the fraction of α-Syn-positive RGCs in response to ONC. Surprisingly, the fraction of α-Syn-positive RGCs remained unchanged after injury (Fig. 5K), indicating that α-Syn positive RGCs is not affected by the traumatic event. As Snca expression showed differential patterns among RGC subtypes, we investigated whether RGCs with varying Snca expression levels displayed differential vulnerability to injury. However, our analysis of high Snca-expressing ipRGCs revealed no changes in α-Syn-positive ipRGCs fractions after injury (Fig. 5F, I, M), suggesting that Snca expression levels do not determine RGC vulnerability to trauma.

### Alterations of cell-cell communication network after ONC

**Figure 7:**
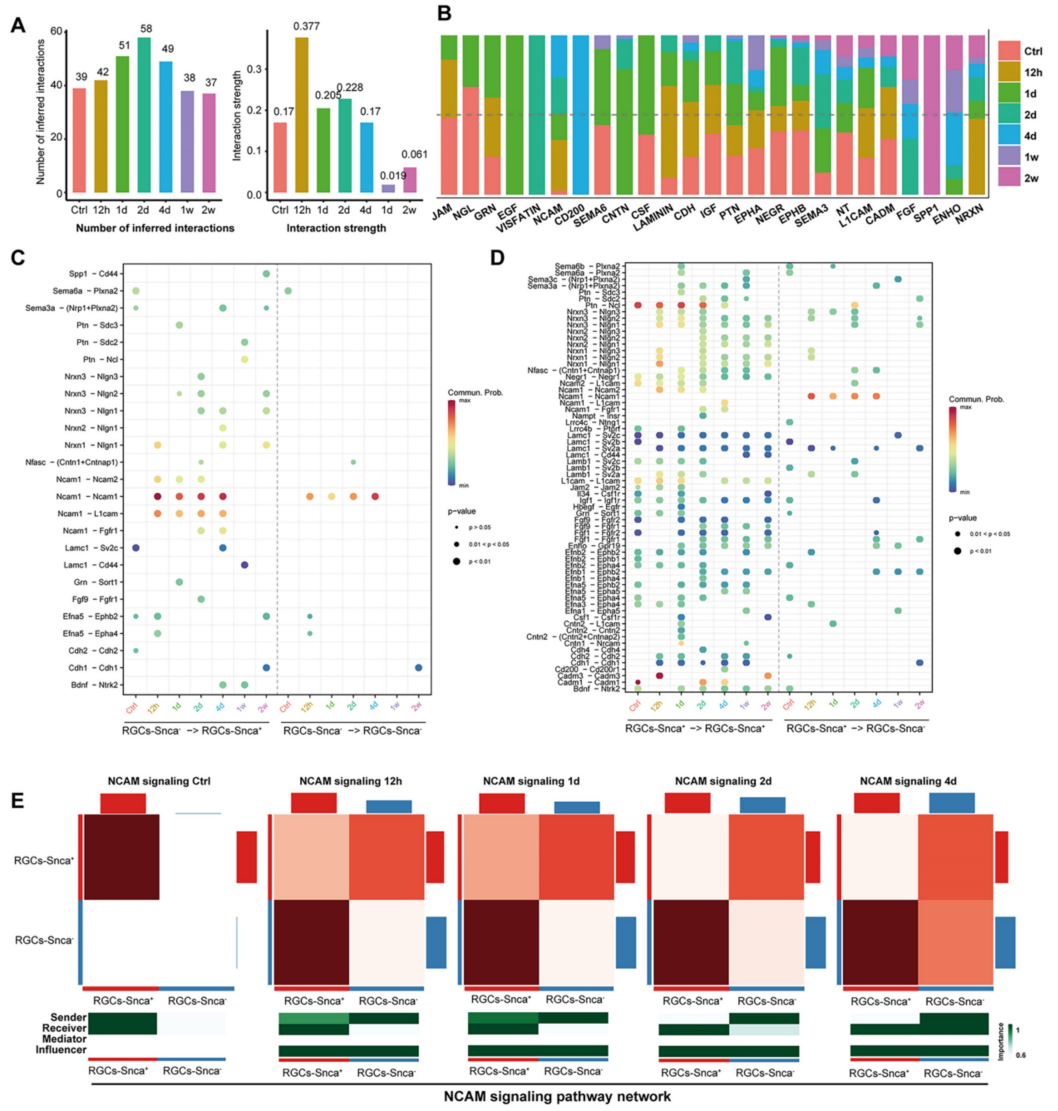
** Cell-cell communication analysis at each time points following ONC. (A)** A bar plots depicts the number of cellular communication (left) and interaction strength (right) in each time points following ONC, respectively. The horizontal axis represents the different time points following ONC. The vertical axis respectively indicates the number of cell communications (left) and the magnitude of interaction strength (right).** (B)** A bar chart displaying the difference in the relative ratio of information flow in the interaction network between each time points following ONC. **(C-D)** The dot plot shows the ligand expressing cell-the receptor expressing cell (horizontal axis) and significant ligand-receptor pairs (vertical axis). The color of the dots represents the calculated interaction probability, and the dark red dot represents stronger predicted interactions. P < 0.05 was considered significant interactions. The dot plot shows the significant ligand-receptor pairs between RGCs-Snca- - RGCs-Snca+ (ligand- receptor) and RGCs-Snca- - RGCs-Snca- at each time points following ONC **(C)**. The dot plot displays the significant ligand-receptor pairs between RGCs-Snca+ - RGCs-Snca+ and RGCs-Snca+ - RGCs-Snca- at each time points following ONC **(D)**. **(E)** The heatmap presents the relative importance of each cell group (RGCs-Snca+ and RGCs-Snca-) based on the computed network centrality measures of NCAM signaling in each time points following ONC, respectively. The intensity of the color on the heatmap corresponds to the strength of interaction between the cell pairs, with darker hues indicating stronger interactions.

The importance of cellular communication networks in disease progression is increasingly recognized 45-47. In the case of ONC, which results in the transection of RGC axons and subsequent RGC death, the changes in cell-cell communication during this process have not been fully explored. To address this, we conducted an analysis, based on the CellChat analysis, to infer potential intercellular communication networks between RGCs-Snca^+^ and RGCs-Snca^-^ after ONC at different time points. The results showed a significant increase in both the total number and intensity of cell-cell communication in RGCs after ONC, followed by a decline after 4 days, indicating a dynamic response to the injury (Fig. 7A). At one week after the injury, although the number of cell-cell communications remained normal, the intensity of communication was notably low, suggesting a potential silenced state of cell communication in RGCs at the late stage of injury (Fig. 7A). Further, the intensity of communication signals decreased as the injury progressed, eventually leading to a silent state (Fig. 7B). Noteworthy, certain signals, such as JAM, NGL, GRN, SEMA6, CSF, IGF, PTN, and NEGR, exhibited reduced communication signal strength after injury, while others, like NCAM, CNTN, ENHO, NRXN, and LAMININ, which were silent or had low activity in the control state, showed activation after injury, indicating their potential involvement in the specific response of RGCs to cellular damage.

**Figure 8:**
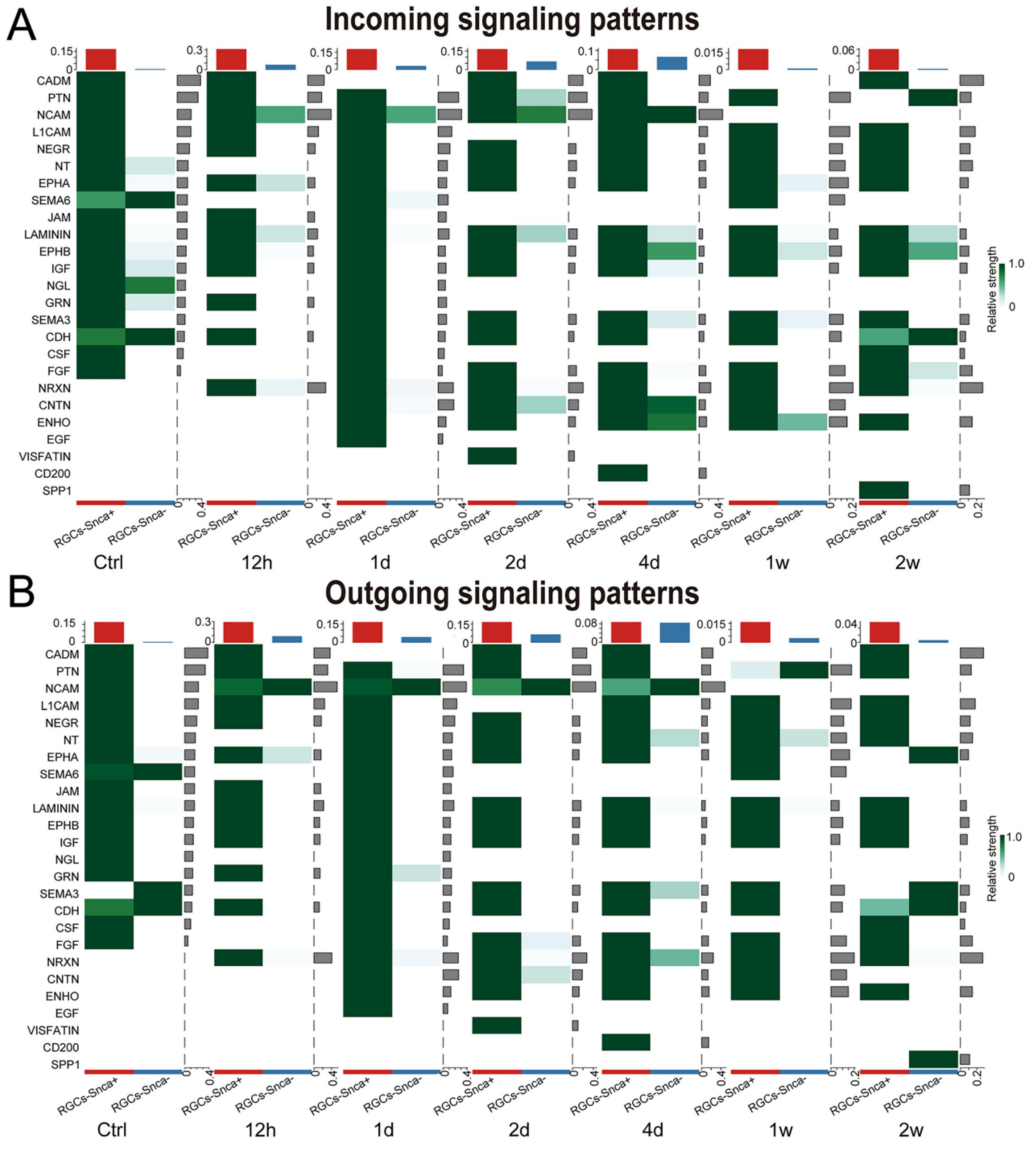
** NCAM signaling was predicted to mediate the interaction between RGCs-Snca+ and RGCs-Snca- at the early stage following ONC. (A-B)** Incoming and outgoing signaling patterns between RGCs-Snca+ and RGCs-Snca- cell populations at each time points following ONC. The ordinate axes represent different signaling pathways. The intensity of the color indicates the strength of the signal, with darker shades corresponding to a stronger signal within the respective signaling pathway in the corresponding cell.

We evaluated incoming and outgoing signaling patterns to further explore the potential signaling interactions between RGCs-Snca+ and RGCs-Snca- at each time point (Fig. 8). Our data indicated that neural cell adhesion molecule (NCAM) signaling may mediate the interaction between RGCs-Snca^+^ and RGCs-Snca^-^ at the early stage following ONC. Furthermore, we peformed a detailed analysis of specific receptor-ligand interactions between RGCs-Snca^+^ and RGCs-Snca^-^ at each time points following the injury (Fig. 7C-D). We found that match probability of receptor-ligand pairs and matched number were abundant in RGCs-Snca^+^- RGCs-Snca^+^ (ligand-receptors) interactions. Notably, the Ptn-Ncl pathway emerged as the most abundant pathway in RGCs-Snca^+^- RGCs-Snca^+^ interactions, while Ncam1-Ncam1 pairs was absent in RGCs-Snca^+^- RGCs-Snca^+^ interactions. The Ncam1-L1cam pairs was specifically observed in RGCs-Snca^-^- RGCs-Snca^+^(Fig.7C-D). Additionally, we further elucidate the network of the NCAM signaling pathway between RGCs-Snca^+^ and RGCs-Snca^-^ following ONC. The data showed that NCAM primarily exhibited self-secretion between RGCs-Snca^+^ under physiological conditions, However, RGCs-Snca^-^ serves as the main sender of NCAM signal and RGCs-Snca^+^ serves as the main signal receiver at the early stage following ONC (Figure 7E). These findings suggest that RGCs-Snca^-^ may serve as the primary sender of NCAM signals to regulate RGCs-Snca^+^, promoting synaptic formation after injury.

## Discussion

### Variability in synuclein expression patterns among retinal cell types

The retina is a complex and diverse tissue, comprising over ~80 distinct cell types, each contributing to specific aspects of visual signal processing 48. The present scRNA-Seq analysis revealed a cell-type specific expression of synuclein family members in the retina, consistent with previous studies at the protein level 2, 3.

Importantly, we observed that this expression pattern is conserved across species, suggesting that synucleins play an indispensable role in visual processing and the overall function of the retina. Specifically, our findings that Snca was highly expressed in RGCs and ACs, and was absent in BCs are in line with our previous IF and immuno-electron microscopy results showing α-Syn IR defined in mouse RGCs and ACs 17. However, our present findings, as well as prior results, deviate somewhat from earlier reports indicating a widespread expression of α-Syn in other species 15. These disparities are likely attributed to variations in the specificity and availability of α-Syn antibodies, as those employed previously are no longer commercially accessible. These may suggest their potential role in the regulation of synaptic communication and signal transmission in RGCs and ACs. Conversely, the absence of Snca in BCs might indicate a different functional significance for these cells in visual processing. Furthermore, Sncb was detected in all retinal neurons in both human and mouse retinas, aligning with previous findings showing β-Syn IR in the GCL, IPL, INL, and OPL in primate and mouse retinas 2, 19. Such widespread expression of Sncb in all retinal neurons suggests its importance in multiple cellular functions, such as synaptic plasticity, neurotransmitter release, and cellular communication within the retina. Lastly, both scRNA-Seq and IF findings demonstrated Sncg as being specifically detected in RGCs, consistent with previous studies reporting γ-Syn as a specific and highly expressed marker in adult mouse RGCs but not in other retinal cell types 20 49. This suggests that Sncg may be crucial for transmitting visual signals from the retina to the brain.

### Differential synuclein expression patterns in RGC subtypes

RGCs are critical components of the retina responsible for transmitting highly processed and integrated visual signals to the brain's visual centers 21, 22. However, RGCs exhibit remarkable diversity, each type having expression of synuclein family members. Our investigation revealed that all synuclein family members were homogeneously expressed across ON, OFF, and ON-OFF RGC subtypes, indicating that synuclein-positive RGCs share similarities in their light response characteristics. Further, based on the classification from Tran *et al.* 28, we found that Sncg is highly expressed in all RGC subtypes, consistent with its role as a marker for RGCs. Similarly, Sncb showed high expression levels in all RGC subtypes, closely resembling Sncg, suggesting that Sncb has the potential to differentiate RGCs within the GCL. In contrast, Snca exhibited differential expression in RGC subtypes. The majority of RGC subtypes were dominated by RGCs-Snca^+^, while only a few RGC subtypes were dominated by RGCs-Snca^-^. These observations indicate distinct gene expression profiles of Snca in RGC populations compared to Sncb and Sncg. The differential expression levels of Snca in various RGC subtypes imply that α-Syn may have distinct and separate roles in the transmission of visual signals from RGCs to the brain. These findings open up new paths of research to explore the specific functions of α-Syn in different RGC subtypes and their contribution to visual signal processing and transmission. To further address these specific roles, we are working on functional analyses in α-Syn knockout and overexpression mice. While direct data has not yet been acquired, these efforts will undoubtedly enrich the scientific community's comprehension of retinal biology. Such insights may prove indispensable for future clinical applications, potentially paving the way for the creation of novel diagnostic methodologies or therapeutic interventions targeting retinal diseases and neurodegenerative disorders.

### Diverse gene expression profiles of synuclein family members in α-RGCs

α-RGCs are a well-studied subtype of RGCs known for their large cell bodies, roarse dendrites and axons, and their broad and monostratified dendritic fields 50. In mice, α-RGCs send their axon terminals to the dorsal lateral geniculate nucleus and superior colliculus, which are primary retinorecipient areas in the brain, playing a crucial role in image processing 51. α-RGCs can be further classified into four subtypes based on their stratify depths in the IPL and their producing transient or sustained responses: On-transient, On-sustained, Off-transient, and Off-sustained 50, 52. Despite prior studies on the morphological, physiological, and molecular properties of these α-RGC subtypes, the gene expression profiles of synuclein family members in α-RGCs and its subtypes remain poorly understood. Our scRNA-seq and IF analyses revealed that α-RGCs highly express β-Syn and γ-Syn but have low expression of α-Syn. Moreover, we observed that Snca was highly expressed in ON-T α-RGCs but had lower expression levels in the other three α-RGC subtypes. Interestingly, the expression patterns of Sncb and Sncg were not significantly different among these four α-RGC subtypes. These findings provide valuable insights into the heterogeneity of α-RGCs and expand our understanding of this cell population. It is important to note that central neuronal somas expressing α-Syn are more vulnerable to degeneration because of the deposition of neurotoxic α-Syn 53. The lack of colocalization between strongly SMI-32 IR somas and α-Syn in α-RGCs suggests that these α-RGCs may not be susceptible to degeneration, particularly in the context of Parkinson's disease (PD) patients and PD mouse models. Consequently, the function of α-RGCs might be minimally affected in the progression of PD.

### High expression of α-synuclein in ipRGC subtypes

ipRGCs are a special type of RGCs, which express the photopigment melanopsin and respond autonomously to light in the mammalian retina. It has been demonstrated that ipRGCs are primarily responsible of non-image forming behaviors of light, such as pupil contraction, circadian rhythm, sleep, and mood management 54. Interestingly, PD patients have non-motor symptoms, in addition to typical motor symptoms, including sleep dysregulation, impairment of the pupillary reflex response, mood disturbances, and circadian rhythms dysfunction 55-58. Correspondingly, evidence shows that α-Syn is highly expressed in neurons of early PD-affected brain regions, such as the olfactory bulb 5. Further, a recent study found that reduced density and complexity of ipRGCs are partially responsible for the dysregulation of these non-motor symptoms 59, 60. The scRNA-seq analysis conducted in our study, along with the previous IF findings 17, corroborate the observation that ipRGCs highly express α-Syn. Additionally, the enrichment of α-Syn in all ipRGC subtypes suggests that these cells may be more susceptible to α-Syn pathology and could play a role in the dysregulation of non-motor symptoms seen in PD. The connection between α-Syn expression in ipRGCs and non-motor symptoms highlights the potential importance of these retinal cells in contributing to the broader manifestations of PD. Further study is necessary to unravel the precise mechanisms underlying the interplay between α-Syn and ipRGCs and to investigate how these cells may contribute to the non-motor symptoms observed in PD patients. Further investigations into the specific roles and functional significance of synuclein family members in α-RGCs and their subtypes carries significant potential for elucidating their contributions to visual processing and their relevance to neurodegenerative diseases. Understanding the vulnerability of distinct RGC subtypes, such as α-RGCs, may inform the development of targeted therapeutic strategies for neurodegenerative disorders impacting the visual system.

### Decreased expression levels of synucleins following axon injury in RGCs

Previous studies have demonstrated the involvement of synucleins in the pathogenesis of ocular neurodegenerative diseases 12, 13, 61, such as glaucoma, characterized by progressive loss of RGCs and axon atrophy, leading to visual field loss 62. Evidence indicates a reduction of synucleins in response to elevated ocular pressure in the retina, optic nerve, and aqueous humor of glaucoma patients and animal models of glaucoma 13, 61, 63. However, the changes in synuclein expression in a mouse model of ONC remain less understood. Our investigation revealed a decrease in the expression of Snca in RGCs following ONC, indicating a correlation between Snca downregulation and reduced RGC loss. These findings underscore the significance of Snca as a potential therapeutic target for optic nerve injury and related diseases. Notably, Teister and colleagues reported that intravitreal injection of α-Syn antibodies significantly improved the RGC survival in the central retina of experimental glaucoma models 64. Furthermore, we observed a decrease in Sncb expression in RGCs post-ONC, consistent with previous reports of Sncb downregulation in different glaucoma animal models 61. Similarly, the reduction in Sncg following ONC aligns with findings from both experimentally induced ONC models and genetic glaucoma mouse model (DBA/2J mouse strain) 12. Additionally, immunohistochemical staining for γ-Syn revealed a partial loss of immunoreactivity in the retinal nerve fiber layer of Alzheimer's disease patients 2. These alteration in synucleins observed in optic nerve injury and glaucoma models suggest their potential relevance to other ocular diseases. Together with the outcomes of our study, these findings support the hypothesis that synucleins may play a pivotal role in the pathophysiology of RGC death in ONC, glaucoma, and other neurodegenerative diseases. Additionally, our analysis revealed that the expression levels of synucleins did not change at 12h and 1d after ONC but dramatically decreased at 2d after ONC. This finding suggests that the 2-day time point after ONC may represent a potential therapeutic window for intervention in cases of optic nerve injury.

### Resilience of α-Syn positive RGCs to traumatic injury

Our comprehensive investigation of the expression pattern of α-Syn in different RGC subtypes provides valuable insights into the susceptibility of specific cell types in the retina. While the function of α-Syn has been extensively studied in the CNS, its physiological roles in the retina have remained largely elusive. Understanding the role of α-Syn in traumatic injury is an essential area of research. In our study, we observed a decrease in α-Syn-positive RGCs following ONC, but interestingly, the fraction of α-Syn-positive RGCs among all RGCs remained unchanged after the injury. This observation suggests that α-Syn may not be highly susceptible to injury in the context of ONC. These findings contrast with the observed RGC loss and reduction in melanopsin-positive RGCs in PD models 59 and α-Syn overexpression in the retina 65, 66. This suggests that α-Syn positive RGCs may not be highly susceptible to traumatic injury but may be more vulnerable to chronic neurodegenerative processes associated with PD. Further research is needed to elucidate the roles of α-Syn in the retina and its potential implications in traumatic and neurodegenerative injury scenarios. Understanding the cell-type-specific vulnerabilities in the retina could have significant implications for the development of targeted therapeutic approaches to protect and preserve visual function in various retinal diseases and injuries.

### Changes in cell-cell communication network following ONC

Our findings revealed that the number and intensity of cell-cell communication increased during the early stage following ONC, but the number of cell-cell communication returned to normal levels during the late stage, while the intensity of cell-cell communication significantly diminished. This suggests that cell-cell communication was heightened in the early stages of ONC, but tended to stabilize in the later stages. We further identified specific signaling pathways that exhibited alterations in response to ONC. The pathways involving JAM, NGL, GRN, SEMA6, CSF, IGF, PTN, and NEGR showed reduced activity, while the pathways involving NCAM, CNTN, ENHO, NRXN, and LAMININ showed increased activity as the injury progressed.

The identification of the Ptn-Ncl pathway as the most abundant in RGCs-Snca^+^- RGCs-Snca^+^ interactions is a significant finding from our analysis. The Ptn gene encodes pleiotrophin, an embryonic neurotrophic protein with increased expression in developing tissues, particularly the nervous system, and it plays important roles in neurite outgrowth and synaptic plasticity 67. The Ncl gene encodes nucleolin, a nuclear protein primarily located in the nucleolus, which transports Ptn into the nucleus to modulate gene expression patterns 67, 68 The enrichment of the Ptn-Ncl pathway in RGCs-Snca^+^- RGCs-Snca^+^ interactions suggests that α-Syn-positive RGCs may engage in specific intercellular communication mediated by these two molecules. Understanding the significance of the Ptn-Ncl pathway in RGCs-Snca^+^- RGCs-Snca^+^ interactions could offer valuable insights into the functional roles of α-Syn in the retina and its potential impact on RGC physiology. Further investigations into the downstream effects of this pathway and its involvement in visual processing and RGC function may shed light on the broader implications of α-Syn expression in the retina and its potential relevance to ocular neurodegenerative diseases.

Of particular interest is the signaling pathway involving PTN, as previous studies have linked the suppression of PTEN expression to enhanced axon regeneration in α-RGCs 30. Given that delaying or blocking the programmed death of RGCs by modulating signaling pathways is crucial in preserving visual function 44, 69, our study provides valuable insights into potential signaling pathways that warrant further investigation in the context of RGC survival and visual dysfunction following ONC. These findings may open new fields for therapeutic interventions aimed at promoting RGC survival and preserving visual function in conditions associated with RGC death, such as glaucoma and optic nerve injuries.

NCAM belonging to the immunoglobulin superfamily, is essential for the nervous system's development and plasticity 70. In our study, we observed the activation of NCAM signals after the injury, which disappeared at 1 week after 2 weeks, contrary to the findings that NCAM decreases drastically within the first 3 days after spinal cord injury and increases thereafter 71. These discrepancies could be attributed to differences in the injury model and approach used in our study. Further, the NCAM signaling pathway exhibited significant communication differences between RGCs with different expression levels of α-Syn. Moreover, the IF quantification results showed a decrease in α-Syn positive RGCs at 1 week following ONC. These findings lead us to speculate that NCAM-mediated pathways might be implicated in the loss of RGCs containing α-Syn. Further experimental evidence is needed to be carried out to support this hypothesis in the ONC model.

In summary, the comprehensive single-cell transcriptome analysis of synuclein family members in healthy retinal cell types and various categorized RGC subtypes, using scRNA-seq, provides novel insights into the heterogeneity of RGCs and their potential vulnerability to α-Syn pathology. Further, our study's innovative approach using scRNA-seq to characterize synuclein expression in retinal cells advances our knowledge of retinal physiology and pathology. This information may pave the way for future research aimed at understanding neuronal vulnerability, retinal function, and the potential development of targeted therapies for ocular neurodegenerative diseases.

## Figures and Tables

**Table 1:** Primary Antibody Characterization

Antiserum	Cat #	Working dilution	Cell type
Mouse anti-α-Synuclein	Abcam, ab1903, RRID: AB_302665	1:1000	α-Synuclein-IR 17
Rabbit anti-α-Synuclein	Abcam, ab212184	1:1500	α-Synuclein-IR 17
Rabbit anti-β-Synuclein	Abcam, ab76111, RRID: AB_1309981	1:1500	β-Synuclein-IR 3
Rabbit anti-γ-Synuclein	Abcam, ab55424, RRID: AB_2193398	1:100	γ-Synuclein-IR 72
Mouse anti-SMI32	Biolegend, 801702, RRID: AB_2715852	1:1000	α-RGCs 22, 73
Rabbit anti-Melanopsin	Advanced Targeting Systems, AB-N39, RRID: AB_1608076	1:3000	ipRGC 74, 75
Rabbit anti-RBPMS	Proteintech, 15187-1-AP, RRID: AB_2238431	1:200	RGCs 65, 76
Mouse anti-RBPMS	Santa Cruz Biotech, SC-293285, RRID: AB_2910236	1:50	RGCs 77, 78
Sheep anti-Chx10	Abcam, ab16141, RRID: AB_302278	1:50	BCs 79, 80
Mouse anti-AP2α	Developmental Studies Hybridoma Bank, AB 528084, RRID: AB_528084	1:800	ACs 81, 82

IR: Immunoreactive; RGCs: Retinal ganglion cells; BCs: Bipolar cells; ACs: Amacrine cells; ipRGC: intrinsically photosensitive retinal ganglion cells; α-RGCs: alpha- retinal ganglion cells.
